# Supplementary material for: Controllable Nitric Oxide Storage and Release in Cu-BTC: Crystallographic Insights and Bioactivity
Source: Int J Mol Sci. 2022 Aug 13;23(16):9098. doi: 10.3390/ijms23169098 (PMC9409197; doi:10.3390/ijms23169098)
Supplement: Supplementary file 1 [file ijms-23-09098-s001.zip › ijms-1834942-supplementary.pdf]

Supporting Information

Controllable Nitric Oxide Storage and Release in Cu-BTC: Crystallographic Insights and Bioactivity

Do Nam Lee [1], Yeong Rim Kim [2], Sohyeon Yang [3], Ngoc Minh Tran [4,5], Bong Joo Park [6,*], Su Jung Lee [1], Youngmee Kim [3], Hyojong Yoo [4], Sung-Jin Kim [3] and Jae Ho Shin [2,*]

1 Ingenium College of Liberal Arts (Chemistry), Kwangwoon University, Seoul 01897, Korea
2 Department of Chemistry, Kwangwoon University, Seoul 01897, Korea
3 Nanobio-Energy Materials Center and Department of Chemistry and Nano Science, Ewha Womans University, Seoul 03760, Korea
4 Department of Materials Science and Chemical Engineering, Hanyang University, Ansan-si 15588, Korea
5 Department of Chemistry, University of Sciences, Hue University, Hue City 530000, Vietnam
6 Department of Electrical and Biological Physics, Kwangwoon University, Seoul 01897, Korea
* Correspondence: parkbj@kw.ac.kr (B.J.P.); jhshin@kw.ac.kr (J.H.S.);
Tel.: +82-2-940-8629 (B.J.P.); +82-2-940-5627 (J.H.S.)

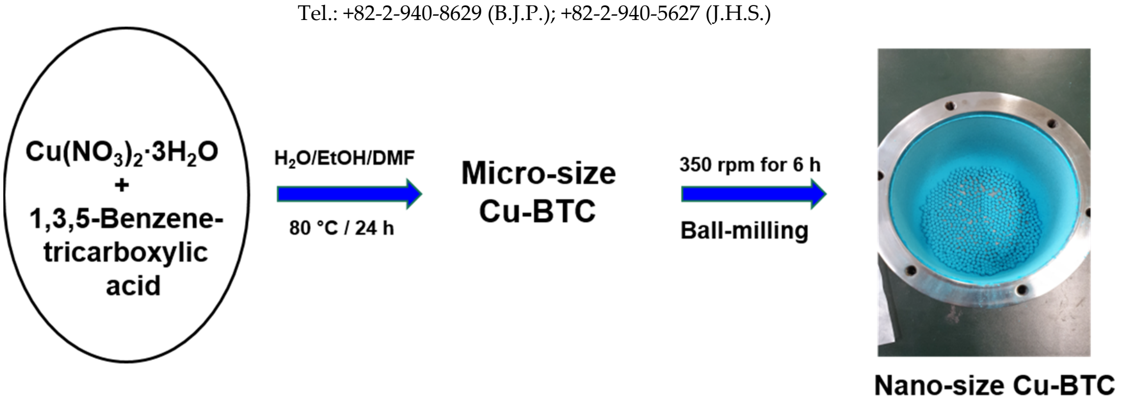

Figure S1. Schematic of the synthesis of micro-sized Cu-BTC (**1**, BTC: benzene-1,3,5-tricarboxylate) by a solvothermal reaction and the preparation of nano-sized Cu-BTC (**2**) by ball milling.

Table S1. Bond lengths [Å] and angles [°] for NO⊂Cu-BTC (NO: nitric oxide).

Cu(1)-O(1)	1.951(4)
Cu(1)-O(1)#1	1.951(4)
Cu(1)-O(1)#2	1.951(4)
Cu(1)-O(1)#3	1.951(4)
Cu(1)-O(2)	2.192(11)
Cu(1)-Cu(1)#4	2.622(3)
O(1)-C(1)	1.260(5)
C(1)-O(1)#5	1.260(5)
C(1)-C(2)	1.492(10)
C(2)-C(3)#6	1.385(6)
C(2)-C(3)	1.385(6)
C(3)-C(2)#7	1.385(6)
C(3)-H(3)	0.9400
N(11)-O(11)	1.150(2)
O(1)-Cu(1)-O(1)#1	89.1(3)
O(1)-Cu(1)-O(1)#2	89.8(3)
O(1)#1-Cu(1)-O(1)#2	168.8(2)
O(1)-Cu(1)-O(1)#3	168.8(2)
O(1)#1-Cu(1)-O(1)#3	89.8(3)
O(1)#2-Cu(1)-O(1)#3	89.1(3)
O(1)-Cu(1)-O(2)	95.59(12)
O(1)#1-Cu(1)-O(2)	95.58(12)
O(1)#2-Cu(1)-O(2)	95.58(12)

O(1)#3-Cu(1)-O(2)	95.58(12)
O(1)-Cu(1)-Cu(1)#4	84.41(12)
O(1)#1-Cu(1)-Cu(1)#4	84.42(12)
O(1)#2-Cu(1)-Cu(1)#4	84.42(12)
O(1)#3-Cu(1)-Cu(1)#4	84.42(12)
O(2)-Cu(1)-Cu(1)#4	180.0(3)
C(1)-O(1)-Cu(1)	122.7(4)
O(1)-C(1)-O(1)#5	125.6(7)
O(1)-C(1)-C(2)	117.2(4)
O(1)#5-C(1)-C(2)	117.2(4)
C(3)#6-C(2)-C(3)	118.6(8)
C(3)#6-C(2)-C(1)	120.6(4)
C(3)-C(2)-C(1)	120.6(4)
C(2)-C(3)-C(2)#7	121.5(8)
C(2)-C(3)-H(3)	119.3
C(2)#7-C(3)-H(3)	119.3

Symmetry transformations used for generating equivalent atoms:

#1 y,x,z; #2 x,y,-z+2; #3 y,x,-z+2; #4 -x+1/2,-y+1/2,-z+2; #5 -y+1/2,-x+1/2,z; #6 -y+1/2,-z+1,x+1/2; #7 z-1/2,-x+1/2,-y+1

Table S2. The nitrogen sorption properties of micro-sized Cu-BTC and nano-sized Cu-BTC.

	**Micro-sized Cu-BTC**	**Nano-sized Cu-BTC**
Specific surface area (m^2 g^{-1})	799	27
Micropore volume (cm^3 g^{-1})	0.3283	0.0092
Mesopore volume (cm^3 g^{-1})	0.0096	0.0570
Mean pore diameter (nm)	1.5234	9.9172
